# Investigation of house dust mite induced allergy using logistic regression in West Bengal, India^☆^

**DOI:** 10.1016/j.waojou.2019.100088

**Published:** 2019-11-27

**Authors:** Priti Mondal, Debarati Dey, Nimai Chandra Saha, Saibal Moitra, Goutam Kumar Saha, Srijit Bhattacharya, Sanjoy Podder

**Affiliations:** a Allergology and Applied Entomology Research Laboratory, Post Graduate Department of Zoology, Barasat Government College, Kolkata, 700124, West Bengal, India; b Department of Zoology, University of Calcutta, Kolkata, 700019, West Bengal, India; c Vice-Chancellor, University of Burdwan, Burdwan, 713104, West Bengal, India; d Allergy and Asthma Research Centre, Kolkata, West Bengal, India; e Post Graduate Department of Physics, Barasat Government College, Kolkata, 700124, West Bengal, India

**Keywords:** Asthma, House dust mite allergy, Logistic regression model, ROC, SPT, HDM, House Dust Mite, SPT, Skin Prick Test, ROC, Receiver Operating Characteristics, IgE, Immunoglobulin E, tIgE, Total IgE, AUC, Area Under the Curve, PPV, Positive Predictive Value, NPV, Negative Predictive Value, RAST, Radioallergosorbent Test

## Abstract

**Background:**

The diagnosis of house dust mite (HDM) allergy based on Skin prick test (SPT) is not accurate, especially in lower risk cases. Our aim is to develop and validate a predictive model to diagnose the HDM allergic symptoms (urticaria, allergic rhinitis, asthma).

**Methods:**

A forward-step logistic regression model was developed using a data set of 537 patients of West Bengal, India consisting of clinical variables (SPT based on 6 allergens of house dust and house dust mites, total IgE) and demographic characteristics (age, sex, house conditions). The output probability was estimated from the allergic symptoms shown by the patients. We finally prospectively validated a data set of 600 patients.

**Results:**

The gradual inclusion of the variables increased the correlation between observed and predicted probabilities (correlation coefficient (r^2^) = 0.97). The model development using group-1 showed an accuracy rate of 99%, sensitivity and specificity of 99.7% and 88.6% respectively and the area under the receiver operating characteristics (ROC) curve (AUC) of 99%. The corresponding numbers for the validation of our model with group-2 were 87%, 95.6% and 66% and 86% respectively. The model predicted the probability of symptoms better than SPTs in combination (accuracy rate 0.76–0.80), especially in lower risk cases (probability< 0.8) that are highly difficult to diagnose.

**Conclusion:**

This is perhaps the first attempt to model the outcome of HDM allergy in terms of symptoms, which could open up an alternative but highly efficient way for accurate diagnosis of HDM allergy enhancing the efficiency of immunotherapy.

## Introduction

House dust mite (HDM) is the commonest of allergens responsible for the pathogenesis of allergic respiratory diseases like allergic rhinitis, urticaria, and, most importantly, asthma. The general treatment strategies for controlling HDM allergic diseases consist of the avoidance of the allergens and symptomatic pharmacotherapy until the symptoms end.^1^, ^2^, ^3^ That the HDM allergy problem could not be resolved completely with the avoidance strategy may need further effective approaches for rapid diagnosis, setting up suitable treatment plans and, above all, to identify the crux of the problem. Inaccurate prognosis of HDM sensitization delays the efficient treatment of the concerned patients. The reasons behind the lack of effective diagnosis and treatment could be the scarcity of the systematic data of HDM patients and their clinical investigations. The recent introduction of allergen-specific immunotherapy^3^ along with different environmental control programs may improve the disease management skill in the near future but the lack of systematic data and clinical evidences could again impede the whole process. These underlying troubles have added to the woes of allergic patients globally. Amazingly, at present, around 235 million population people worldwide have been suffering from asthma, a chronic respiratory disease, mostly resulting from the HDM allergy.^4^ Despite the modernization of medicines and bronchodilators, asthma cannot be controlled, and the occurrence of asthma continues to increase.

For house dust mite allergy diagnosis, skin prick test (SPT) is vital and considered as the pillar stone of HDM allergy diagnosis.^5^, ^6^, ^7^ But only the SPT or sIgE cannot predict HDM allergy accurately, especially the extent of the disease. In other diseases, where diagnosis is highly difficult, mathematical models have been found quite successful for prediction.^7^, ^8^, ^9^ Logistic regression model has been used regularly to study the risk assessment of different diseases. The model is capable of selecting the independent clinical and demographical predictors required for accurate diagnosis. This simple yet highly powerful approach has successfully been applied in Hepatitis B and liver fibrosis patients.^10^^,^^11^ Again, DunnGalvin et al.^12^ have constructed a simple formula, based on forward logistic regression, incorporating age, sex, clinical history, SPT, total IgE (tIgE), etc., predictors as clinical variables, which has successfully predicted (95% accuracy) the food allergy. But in the field of HDM allergy such attempts were never witnessed. Lack of available data could possibly be the major reason. This prompted us to record demographic characteristics (age, sex, residential house condition, etc.) of 1137 patients in India and the clinical results of their SPT, total IgE along with the histories of asthma, urticaria and allergic rhinitis. Then we propose a simple logistic regression formula to predict the probability of the HDM allergic symptoms. The development of such predictive models would aid researchers in the diagnosis and risk assessment of suspected HDM allergic diseases and to formulate future strategies.

## Materials and methods

### Study group

The current study includes 1137 clinically diagnosed nasobronchial allergic patients attending the out-patient department of Allergy and Asthma Research Center, Kolkata, India between October 2016 to November 2018. Patients were provided with a questionnaire to fill up the demographic information including age, sex, house condition, and clinical history.

### Skin prick test (SPT)

Allergic SPT was performed^13^^,^^14^ on the participating individuals with allergen extracts consisting of house dust (SPT1) and 5 house dust mites, namely SPT2 (*Dermatophagoides farinae),* SPT3 *(Dermatophagoides pteronyssinus),* SPT4 (*Acarus siro),* SPT5 (*Lepidoglyphus destructor),* and SPT6 (*Tyrophagus putrescentiae)*. The allergen extracts were supplied by MERCK. The house dust extract was supplied for positive controls and negative controls by Credisol ® Mumbai, India. 10 mg/ml of histamine phosphate and 0.9% sterile saline were used, respectively. Wheal diameter was recorded after 15 minutes of the test performed. An allergen eliciting ≥3 mm wheal diameter was considered as positive SPT.

### Total IgE (tIgE)

For total IgE estimation in serum, peripheral intravenous blood samplings of the patients were done. Enzyme-linked immunoassay was then performed using chemicals from Pathozyme Immunoglobulin (Ref. OD 417) supplied by Glaxo Smithkline Pharmaceuticals Ltd, Mumbai.

### Predictors

To construct a logistic regression model we use the following predictors: sex, age, house condition, SPT for house dust allergen, SPT for 5 different HDM allergens as mentioned earlier, and total IgE (tIgE).

Scores of 1 and 0 were given to male and female patients, respectively. Since the HDM allergy was already found to depend on house conditions,^15^ the "dry and new house" has been subjected to a score of 0, while the "humid and old" one is marked as 1. According to the allergic symptoms like allergic rhinitis, urticaria, and asthma, the patients have been given a score from 0 to 7. The rules of score selection according to the severity of symptoms closely correspond to past literature,^16^ and they have been shown in Table 1. This score, normalized by the highest value, was treated as the probability of HDM allergy outcome. Normalization was performed so that the probability could remain within the range 0–1. The values of SPT, total IgE, as measured, were taken directly in the calculation.

**Table 1:** Demographic and clinical characteristics of first group

n = 537	Characteristics	Mean ± S.D	Regression coefficient (95% CI)
Sex	Male = 239		0.045 ± 0.014
Age (years)	4–78	35 ± 16	0.0055 ± 0.0012
SPT1 (mm)	1.00–6.80	3.39 ± 1.10	0.690 ± 0.180
SPT2 (mm)	1.00–8.10	2.51 ± 1.53	0.75 ± 0.11
SPT3 (mm)	1.00–10.50	2.34 ± 1.31	0.74 ± 0.11
SPT4 (mm)	1.00–4.40	2.32 ± 0.79	0.70 ± 0.08
SPT5 (mm)	1.00–4.20	2.28 ± 0.67	0.70 ± 0.08
SPT6 (mm)	1.00–5.60	2.26 ± 0.71	0.75 ± 0.12
Total IgE(kUa/L)	5.0–7448.0	570.7 ± 976.2	−0.00015 ± 0.00008
Symptoms	Number of patients	Scores	
No symptoms	5	0	
Allergic rhinitis	53	1	
Urticaria	67	2	
Asthma	56	3	
Rhinitis and urticaria	65	4	
Urticaria and asthma	60	5	
Rhinitis and asthma	134	6	
Rhinitis and urticaria and asthma	97	7	
Probability of diagnosis	0.00–1.00	0.49 ± 0.28	

### Data analysis

Two groups were formed randomly, to eliminate potential confounders (Wunsch 2007),^17^ with 537 and 600 patients for the model development and validation purposes, respectively. We developed a multivariate stepwise forward logistic regression model as per the following formula (equation (1)) to calculate the probability of HDM allergy (π). The logistic regression model has been developed or trained using the data of group 1 (shown in Table 1). The model developed such a way has finally been used to predict the probability of symptoms for group 2 (Table 2).

**Table 2:** Demographic and clinical characteristics of second group.

n = 600	Characteristics	Mean ± S.D
Gender	Male = 328	
Age (years)	6–78	35 ± 15
SPT1 (mm)	1.00–6.80	3.41 ± 1.12
SPT2 (mm)	1.00–8.10	2.54 ± 1.02
SPT3 (mm)	1.00–10.50	2.38 ± 1.19
SPT4 (mm)	1.00–4.40	2.32 ± 0.77
SPT5 (mm)	1.00–4.20	2.27 ± 0.66
SPT6 (mm)	1.00–5.60	2.24 ± 0.70
Total IgE(kUA/L)	18.0–7449.0	601.5 ± 984.4
Symptoms	Number of patients	Scores
No symptoms	4	Described in Table 1
Allergic rhinitis	67	
Urticaria	74	
Asthma	62	
Rhinitis and urticaria	73	
Urticaria and asthma	66	
Rhinitis and asthma	63	
Rhinitis and urticaria and asthma	108	
Probability of diagnosis	0.00–1.00	0.63 ± 0.29

SPT1:house dust, SPT2: Dermatophagoides farinae, SPT3:Dermatophagoides pteronyssinus, SPT4: Acarus siro, SPT5: Lepidoglyphus destructor, SPT6: Tyrophagus putrescentiae

Logistic regression model is generally used in the calculation where the outcome is discrete and has limited number of values. In our case, the HDM allergy probability, depending on the severity of the symptoms, is discontinuous, ranging broadly between 0 to 1. The logistic regression depends on logistic function having sigmoid in shape also ranging between 0 to 1. For multivariate logistic regression model, the probability becomes, $\pi =\frac{1}{1+\exp\;\left(t\right)}$ , where *t = a* + *bx*_*1*_ + *cx*_*2*_ + *dx*_*3*_ + *….….….*; *b,c,…* are the coefficients of predictor variables *x*_*1*_*, x*_*2*_*, …*etc and *a* is the constant term*.*

The final equation is given by,

(1) $$\pi =\frac{\exp\;\left[-7.65+\left(0.0055\times age\right)+\left(0.045\times sex\right)+\left(0.690\times SPT1\right)+\left(0.75\times SPT2\right)+\left(0.74\times SPT3\right)+\left(0.70\times SPT4\right)+\left(0.70\times SPT5\right)+\left(0.75\times SPT6\right)+\left(-0.00015\times tIgE\right)+\left(0.4\times house\right)\right]}{\left\{[1+\exp\;\left[-7.65+\left(0.0055\times age\right)+\left(0.045\times sex\right)+\left(0.690\times SPT1\right)+\left(0.75\times SPT2\right)+\left(0.74\times SPT3\right)+\left(0.70\times SPT4\right)+\left(0.70\times SPT5\right)+\left(0.75\times SPT6\right)+\left(-0.00015\times tIgE\right)+\left(0.4\times house\right)\right]\right\}}$$

Stepwise regression model has the capability to choose the predictive variables and to produce best possible results in an automated procedure.^12^^,^^18^ In our case, we initiated forward step regression with no predictive variables but with one intercept only. Then different predictive variables have been superposed within a single model each having one regression co-efficient. The best value of the regression coefficients has been estimated in our model individually using univariate logistic models by comparing with the observed probability of symptoms for best value of the correlation coefficient (r^2^). Inclusion of new variables has been continued and finally terminated until the correlation coefficient had not increased significantly (>.005). SYSTAT made SIGMA PLOT 10 was used for all the statistical analysis.

## Results

The age of the first group varied between 4 to 78 years with mean age of 35 years (standard deviation or SD = 16 years). The males and females were in the ratio of approximately 1.24:1. But no significant differences existed between the symptoms of the male and female patients (t = 0.56, P = 0.90). Similarly, no significant differences existed between male and female SPT wheal sizes (t = 0.53, P = 0.296).

The age varied between 6 to 78 years with mean age of 35 years (standard deviation 15 years) for the second group. The males and female patients were in the ratio of almost 1.09:1. Similar to the previous group, no significant differences existed between the symptoms of the male and female patients (t = 0.58, P = 0.80). The trend was also found similar for the SPT wheal sizes and total IgE.

**Fig. 1:**
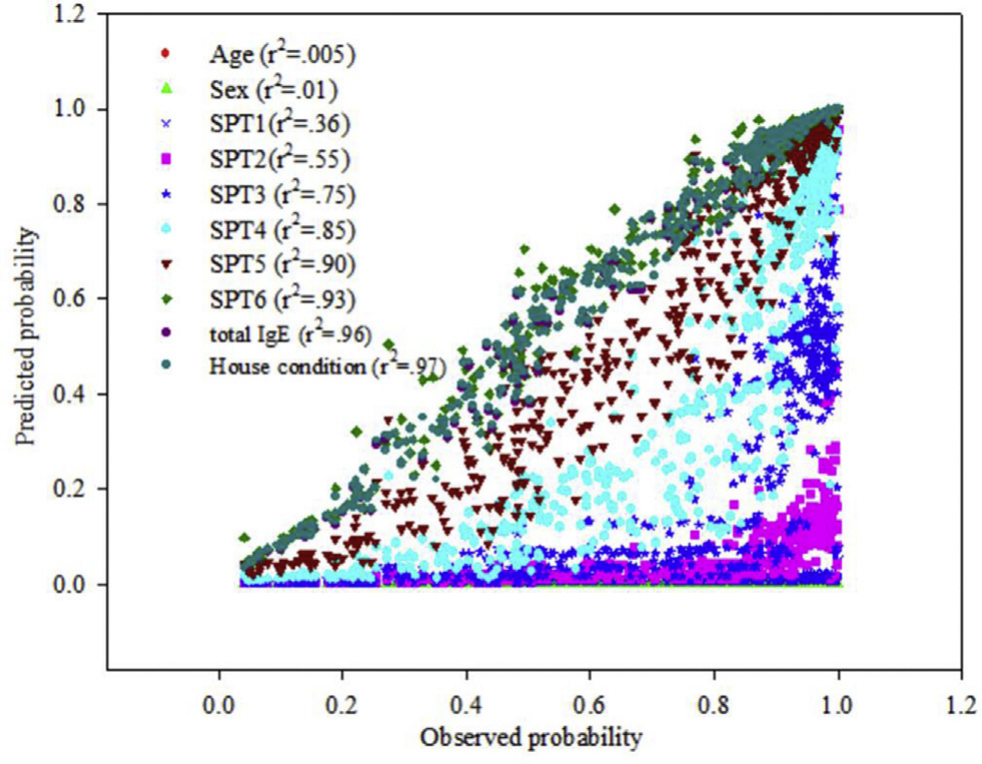
The calibration of forward stepwise logistic regression model. Correlation coefficient (r^2^) increases as new variables are added individually.

**Fig. 2:**
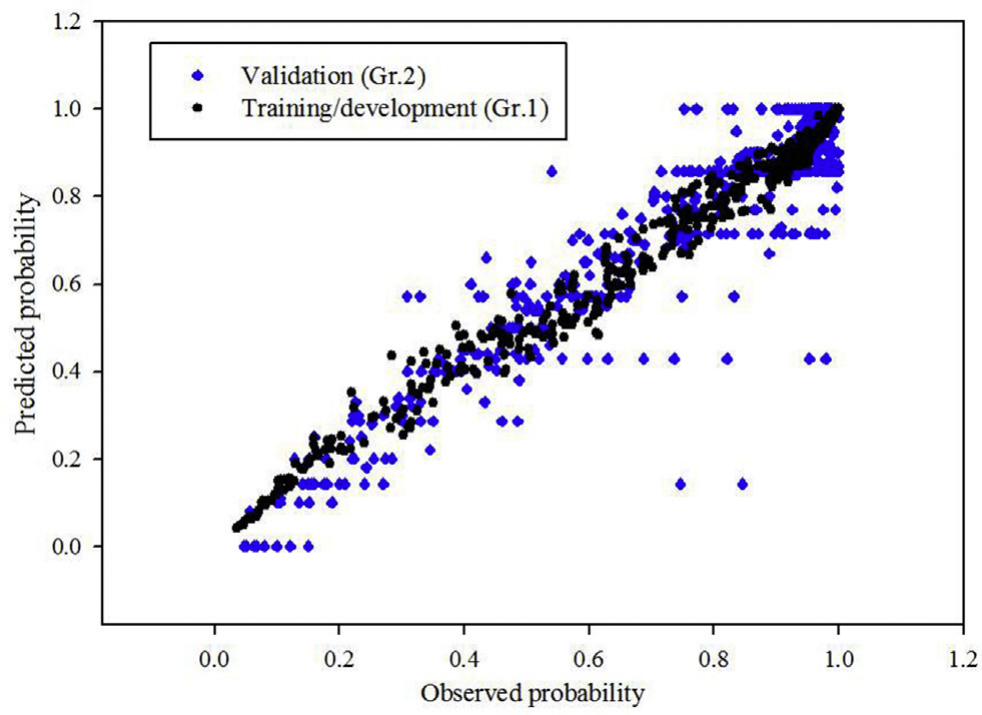
The predicted and observed probabilities of symptoms for groups 1 (black filled circles) and 2 (blue filled inverted triangles).

The calibration of the logistic model comparing the predicted probability of symptoms and observed probability has been shown in the Fig. 1. The variables have been added in the multivariate model one by one and the performance improved subsequently. The improvement has been evident in the *r*^*2*^ value shown in the graph. The best calibration was finally achieved with *r*^*2*^ = 0.97. The corresponding Hosmer-Lemeshow test showed a P value of 0.98. The predicted vs. observed probability for the second group (P value of Hosmer-Lameshow test is 0.88) along with the first group are shown in Fig. 2.

**Fig. 3:**
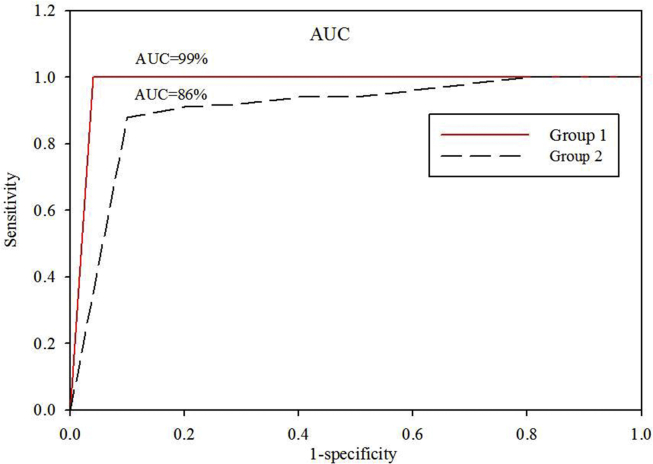
ROC curves for group 1 (red continuous) and group 2 (black dashed curve). The corresponding AUC is also shown in percentage.

The model performance is tested from the receiver operator characteristics (ROC) curve, accuracy rate, sensitivity, specificity, positive predictive value (PPV), and negative predictive value (NPV). The AUC or area under the ROC curve (Fig. 3) was found 0.99 (95% CI, 0.92–0.99) for the first group. This AUC of sensitivity vs. (1-specificity) curve shows the quality of the model prediction. AUC nearer to 1 makes the model good, while AUC nearer to 0.5 makes the model worse.^12^ In this study, if the prediction lied within 1 standard deviation (sd) from the observed value, we assumed the prediction as true (either positive or negative). Whereas the prediction greater than 1sd has been assumed as false.

For the estimation of accuracy rate of the total training set, the cutoff value of the probability of the symptom was taken as 0.5. The confusion matrix is shown in Table 3 for this case with an accuracy rate of 99%. For the cutoff of 0.6 the accuracy rate was found quite similar (98.8%). Other quantities like sensitivity, specificity, etc., are also shown in Table 3.

**Table 3:** Logistic regression model prediction features.

Group 1	Observed		FNR	FPR	Sensitivity/TPR	Specificity	ppv	npv	accuracy	cutoff
Predicted	395 (tp)	1 (fn)	0.003	0.113	99.7	94.6	0.96	0.99	99%	0.6
	16 (fp)	125 (tn)								
Predicted	406 (tp)	1 (fn)	0.01	0.050	99.6	95.4	0.98	0.99	98.8%	0.5
	5 (fp)	126 (tn)								
Group 2	Observed									
Predicted	400	18	0.091	0.044	95.60	65.9	0.97	0.87	87%	0.6
	62	120								
Predicted	398	23	0.055	0.336	94.50	66.4	0.87	0.84	86%	0.5
	60	119								

tp: true positive, fn: false negative.

fp: false positive, tn: true negative.

FNR: False negative rate, FPR: False positive rate, TPR: True positive rate, ppv: positive predictive value, npv: negative predictive value

The model has been validated with the data of group 2 giving AUC = 0.86 (95% CI, 0.81–0.89). The accuracy rate was found 87% for a cutoff of 0.5 and 86% for a cutoff of 0.6. The corresponding confusion matrix is shown in Table 3.

The accuracy rate of the prediction using only the six SPT variables was found much lower (in between 0.76 to 0.78) than that of logistic model. The correlation coefficient (r^2^) varied between 0.51 to 0.67 in case only SPTs were used as predictive variables. Besides, SPT as a predictor performed well if the probability of symptoms were greater than 0.85 but below 0.85 the performance weakened severely. Therefore, for lower risk cases logistic regression model is found better suited than the SPT. Similar trend of logistic models has also been found in the diagnosis of food allergy outcomes given in ref 18. Even if we combine anamnesis data with the SPT variables, the accuracy rate did not increase.

In our study, the males had shown more symptoms than the females. For probability greater than 0.5, the ratio of the symptoms in male and female came nearly to 1.20:1 [F = 1.05, 95% CI=(0.76:1.30), P = 0.56]. For probability less than 0.5, the ratio was 1.26:1 [F = 0.93, 95% CI= (0.79,1.26),P = 0.54].

## Discussion

HDM allergy has a serious influence on the quality of life of a person. Sensitization to house dust mite can preliminarily be diagnosed by SPT and finally by RAST test (Radioallergosorbent test) or histamine release test. However, SPT cannot conclusively prove HDM allergy. In this work, we have constructed a simple logistic regression model-based formula, incorporating both the important clinical and demographic variables that can successfully predict HDM allergy, even in lower risk cases. In some diseases logistic regression model has been used in the past to predict the outcomes. But very few of them validated themselves with new data sets.^12^ On the contrary, our model has been trained with a data set (n = 537) and validated with another new data set (n = 600). We have shown that the judicious choice of variables can significantly increase the predicting probability of a model, which is the advantage of using a forward step regression method.

The complication of the treatment of HDM allergy was described elsewhere.^19^ The avoidance is the best possible control measure until now to prevent HDM allergy.^20^ The measurement of SPT, RAST, histamine release test could diagnose the disease. However we have found that the SPT cannot be used comprehensively for risk assessment, especially lower risk cases for probability π < 0.85. Although the avoidance has also been found useful in many cases such as targeted multiple avoidance measures to avoid asthma in atopic children yet the efficacy of avoidance has also been questioned repeatedly. Unfortunately, this avoidance strategy, either single or multiple, suffers from heterogeneity in clinical trial design, end point definition, and validated outcomes.^3^ Under these circumstances, the need of predictive model is highly important in the field of HDM allergy not only for perfect diagnosis but also for correct pharmacotherapy and the selection of the exact time duration of treatment. Our model does not need rigorous search for house dust mites in the residence of a patient, nor does it need molecular allergy diagnostics, at least initially, to predict the condition of a patient. However, the accuracy rate of the model prediction could be increased if more relevant symptoms and variables had been studied and added in the input of the model.

Recent introduction of immunotherapy can be frustrating in case of HDM allergy than pollen or other allergies.^21^This could be due to the lack of the finding of major HDM allergens or lack of data of HDM allergic patients. The success rate of immunotherapy can be increased if more data are available. Again, the use of simple mathematical predictive model may also improve the performance of immunotherapy for HDM.

Our model predicted almost 97% of positive and 100% of negative cases correctly for group 1. For group 2, they were 87% in case of both the positive and negative. If SPT is used alone, the accuracy rate could come within 0.76–0.80, whereas the accuracy rate of our model varied between 0.86 to 0.88. The inclusion of total IgE could not change the accuracy rate significantly. Therefore, demographic factors are also important to increase the prediction capability of HDM allergy. Similar behavior has also been observed in the field of food allergy.^18^

We observed that the sensitivity and specificity for group 1 had been very high but specificity decreased (from 88% to 66%) in group 2. Specificity of test is actually the proportion of people without having the disease. Lower specificity in group 2 implies the inability of the validation test to identify negative results. The specificity could be increased if more and more different data sets are trained and validated with this model, that will, in turn, correct the values of the regression coefficients dynamically. Though, higher sensitivity and lower specificity may also be resulted from over-fitting or under-fitting the calibration curve. In general, high sensitivity tests may have low specificity. Such examples are mammogram, biopsy, etc., where the sensitivity depends on tumor size, patient age, and other factors.^22^ However, SPT for HDM allergy is not related to such factors. The inherent reason of lower specificity, therefore, may be an artifact of data fitting. In any case, to remove the discrepancies in the data fitting we need to revalidate the model with different datasets.

Another important aspect of this work was that all the patients were originated from West Bengal, a state in the eastern part of India. West Bengal, having mostly tropical climate, is an HDM allergy prone area. Therefore, the construction as well as successful validation of a logistic regression model based on the data of such an area is one of the most important features of this study. The allergy to dust mite is a global health problem recognized by the World Health Organization, which affects millions of people around the world. However, this initiative is perhaps the first to predict the HDM allergy, and we propose this to be an alternative diagnostic tool for clinicians, not the ultimate one.

Our study includes people of different ages, sexes, and house conditions. Variable symptoms are also found in the medical histories of the patients. Recent studies have shown that the demographic factors such as age, sex, and house condition also drive HDM allergy; hence, they are taken as variables in this study. Age has recently been found acting as a significant precursor of HDM allergy. The weak lung function test and increased inflammation may increase the extent of allergic sensitization.^23^ Similarly, recent studies have highlighted sex-dependent HDM allergic sensitization. However, the results conflict with each others regarding male or female predominance.^24^ Besides, different studies have emphatically described the prevalence of urticaria, allergic rhinitis, and asthma as symptoms in HDM allergic patients.^25^

However, this model should be revalidated among different geographical and ethnic groups for further clinical inferences. Within this limitation, this work is a firm step towards the formulation of the risk assessment, control, and management strategies of HDM allergy.

In conclusion, this model based on logistic regression is an effective, low cost, and simple approach to tackle HDM allergy that is difficult to be diagnosed clinically. Instead of using only the clinical variables, different non-clinical demographic variables are also utilized in this model. The model has successfully been developed from the data of 537 patients and validated using the data of 600 patients. The accuracy rate in the training has been found as 99%, whereas for validation the accuracy rate was 87%. The perfection in the accuracy can be achieved if more and more data could be used for model validation. The logistic regression model, as with other diseases, can improve the efficacy in the diagnosis and risk assessment of HDM allergy.

## Declarations

### Statement of ethics

Written consent of participation was obtained from the patients and the parents in case of minor. Clinical Research Ethics committee of Allergy and Asthma research Center, Kolkata approved this study (Approval No: CREC-AARC, Ref: 009/15).

### Consent for publication

All the authors confirm their consent for publication.

### Availability of data and materials

Users were guaranteed anonymity, so raw data or individual user data are available.

### Disclosure Statement

The authors have no conflicts of interest to declare.

### Funding sources

Funding was provided by the Department of Biotechnology, Government of West Bengal (sanction No. 568 [Sanc.]/BT-Estt/RD-19/2015 dated August 03, 2016) to Sanjoy Podder.

### Author contributions

Collection, compilation of data and preparation of the first draft of the manuscript was done by Priti Mondal and Debarati Dey. Saibal Moitra diagnosed the patients and provided the sample. Statistical analysis was done by Srijit Bhattacharya and Sanjoy Podder. Nimai Chandra Saha, Goutam Kumar Saha, Srijit Bhattacharya and Sanjoy Podder conceived the study design. Sanjoy Podder supervised the work and prepared the final draft.
